# Transcriptomic Profiling of Adherent Cells Isolated from Wedged Pulmonary Arterial Blood of Patients with Pulmonary Arterial Hypertension Reveals a Predominantly Myeloid Rather than Endothelial Phenotype

**DOI:** 10.3390/ijms27198799

**Published:** 2026-10-01

**Authors:** Nadezhda Boyarskaya, Polina Kuchur, Pavel Docshin, Olga Skortsanu, Daria Smirnova, Natalia Goncharova, Elizaveta Korobchenko, Kirill Lapshin, Olga Moiseeva, Anna Malashicheva

**Affiliations:** 1 Almazov National Medical Research Centre, 2 Akkuratova St., 197341 Saint Petersburg, Russia; boyarskaya_nv@almazovcentre.ru (N.B.); skortsanu_oi@almazovcentre.ru (O.S.); goncharova_ns@almazovcentre.ru (N.G.); korobchenko_em@almazovcentre.ru (E.K.); lapshin_kb@almazovcentre.ru (K.L.); moiseeva_om@almazovcentre.ru (O.M.); 2 Institute of Cytology of the Russian Academy of Sciences, 4 Tikhoretsky Ave., 194064 Saint Petersburg, Russia; kuchurp@incras.ru (P.K.); smirnovadv@incras.ru (D.S.); malashicheva@incras.ru (A.M.)

**Keywords:** pulmonary arterial hypertension, myeloid angiogenic cells, monocyte-derived cells, circulating endothelial cells, transcriptomics, vascular inflammation, matrix metalloproteinases, right heart catheterization

## Abstract

Pulmonary arterial hypertension (PAH) is characterized by progressive vascular remodeling and immune dysregulation. Myeloid angiogenic cells (MACs) are blood-derived mononuclear cells that acquire endothelial-like features at sites of vascular injury, but their transcriptomic phenotype in the pulmonary vascular compartment is poorly characterized. We isolated adherent cells from blood aspirated from the wedged pulmonary artery of four patients with severe PAH during right heart catheterization. The cells were vWF-positive and ACTA2-negative by immunocytochemistry and co-expressed canonical endothelial (*CDH5*, *PECAM1*, *ERG*, *FLT1*) and myeloid (*PTPRC/CD45*, *CD14*, *SPI1*, *IRF8*) transcripts, consistent with MAC-like adherent cultures rather than a conventional endothelial population. Bulk RNA sequencing of these cultures was compared with human pulmonary artery endothelial cells (HPAECs) and HUVECs used as endothelial reference cultures. Relative to both references, PAH MACs showed higher transcript levels of cytokines (*IL1A*, *TNF*, *IL15*, *IL10*) and matrix metalloproteinases (*MMP8/9/12*); relative to HPAECs, they showed lower levels of Notch-associated genes (*DLL4*, *HEYL*). Protein–protein interaction analysis identified modules centered on complement components, myeloid immune receptors and CC chemokines. Because MACs from non-PAH subjects were not available, these differences describe the phenotype of PAH-derived MACs relative to endothelial reference cells rather than a PAH-specific effect. Adherent cultures obtained from wedged pulmonary arterial blood are therefore dominated by a mononuclear phagocyte transcriptional program, which has direct implications for the design of patient-derived pulmonary vascular cell models.

## 1. Introduction

Pulmonary arterial hypertension (PAH) is a severe and progressive disorder of the pulmonary microvasculature, characterized by elevated pulmonary vascular resistance, right ventricular failure, and a high mortality rate despite modern therapeutic advancements [1,2]. The pathobiology of PAH is driven by a complex interplay of vasoconstriction, obliterative vascular remodeling, and in situ thrombosis, where pulmonary artery endothelial cells (PAECs) play a central, initiating role [3,4]. In response to chronic stress, PAECs undergo a profound phenotypic switch, losing their quiescent, barrier-maintaining properties and acquiring a hyperproliferative, apoptosis-resistant state that ultimately leads to the formation of characteristic occlusive plexiform lesions [4,5].

A growing body of evidence highlights that this profound endothelial dysfunction is intrinsically linked to persistent immune dysregulation and chronic inflammation [4,6]. The pulmonary vascular microenvironment in PAH is marked by the heavy infiltration of macrophages, dendritic cells, and pathologic neutrophils, along with elevated circulating levels of pro-inflammatory cytokines and chemokines [4,7]. Within this milieu, endothelial cells act not merely as passive targets of inflammation but as active immunomodulators that orchestrate leukocyte recruitment and perpetuate vascular injury [4,8]. Furthermore, under the influence of metabolic stress, inflammatory cytokines and aberrant signaling pathways, endothelial cells frequently undergo endothelial-to-mesenchymal transition (EndoMT) and senescent phenotypic switching [4,9]. This transition, driven by factors such as dysregulated juxtacrine Notch signaling, exacerbates pathological matrix deposition and structural remodeling of the pulmonary arteries [3].

Despite significant progress in understanding the molecular underpinnings of PAH, translating these findings into precision medicine remains severely hindered by the inaccessibility of the pulmonary vasculature in living patients. Current multi-omic insights predominantly rely on end-stage lung tissues obtained post-mortem or during transplantation [10] or on single-cell atlases of the whole lung [11], which may fail to capture the dynamic, early-stage molecular events of the disease. While in vitro primary cell models provide vital mechanistic data, there is a critical need for accessible, patient-derived endothelial models. Recent pioneer studies have demonstrated the feasibility of isolating PAECs from the balloon tips of Swan-Ganz catheters during routine right heart catheterization (RHC) [6,12]. However, this method heavily relies on mechanical adherence, frequently yields inconsistent results, and may inadvertently select for highly proliferative subpopulations. Moreover, circulating endothelial cells (CECs) have recently emerged as critical non-invasive targets and biomarkers reflecting continuous vascular injury and detachment in pulmonary vascular diseases [13,14].

To address this gap, the present study characterized the transcriptomic and immunophenotypic profile of adherent cells obtained from blood aspirated from the wedged pulmonary artery of patients with PAH during RHC. This sampling site was chosen because it is the only point of the pulmonary arterial circulation that is routinely accessible in living patients, and because blood aspirated during balloon wedging is drawn from the vascular segment immediately upstream of the remodeled microvasculature. Historically referred to as early endothelial progenitor cells, these mononuclear-derived adherent cells are now recognized as myeloid angiogenic cells (MACs), a population that acquires endothelial-like features and supports vascular repair mainly through paracrine mechanisms. Circulating angiogenic cells in PAH have previously been characterized functionally, through migration, adhesion and tube formation assays [15,16], and circulating endothelial cells have been profiled from peripheral venous blood [14]. To our knowledge, however, no genome-wide transcriptomic description of adherent cells obtained directly from the pulmonary arterial compartment has been reported. We therefore performed bulk RNA sequencing of these cultures and compared them with pulmonary artery endothelial cells and HUVECs as endothelial reference populations, with the primary aim of determining which cell population this widely used isolation approach actually yields and which transcriptional programs it carries.

## 2. Results

### 2.1. Isolation and Phenotypic Characterization of Adherent Cells from Wedged Pulmonary Arterial Blood

**Figure 1:**
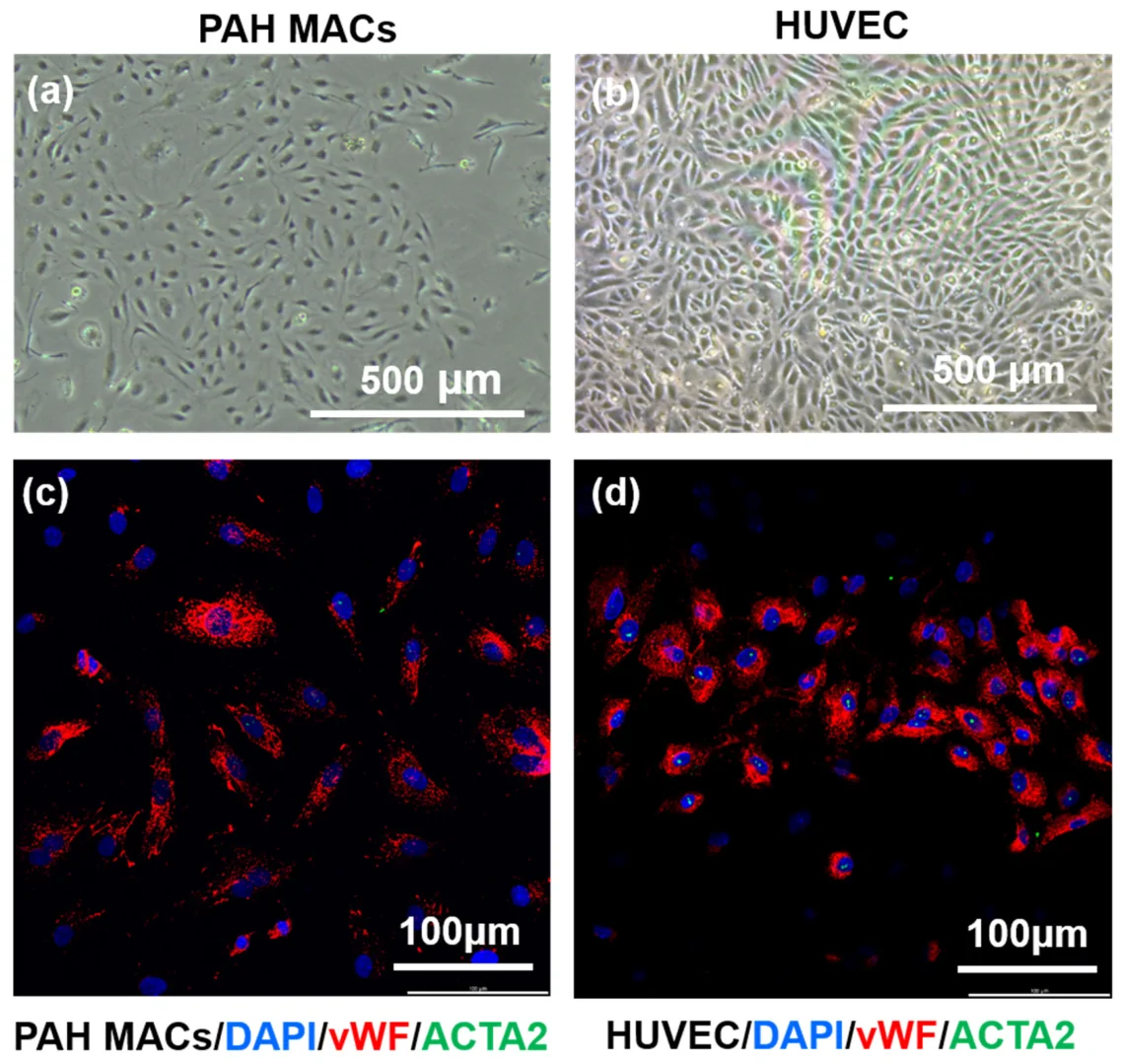
Morphological and immunophenotypic characterization of adherent cells isolated from wedged pulmonary arterial blood (PAH MACs) and reference HUVECs. (**a**,**b**) Representative phase-contrast microscopy images (Nikon Eclipse Ts2 FL, Nikon, Tokyo, Japan; 40× magnification) of (**a**) PAH MACs isolated from blood obtained during right heart catheterization on days 16–20 of culture, and (**b**) reference HUVECs. (**c**,**d**) Confocal fluorescence microscopy images of (**c**) PAH MACs and (**d**) HUVECs stained for von Willebrand factor (vWF, red) and alpha-smooth muscle actin (ACTA2, green); nuclei were counterstained with DAPI (blue). vWF showed the punctate perinuclear distribution characteristic of Weibel-Palade bodies in both cultures, whereas no ACTA2 signal above background was detected in the green channel. Separate single-channel images (DAPI, vWF, ACTA2) and the merged image are provided in Figure S6. Scale bars: 500 μm (**a**,**b**), 100 μm (**c**,**d**).

To investigate the in vitro molecular phenotype of the circulating pulmonary vascular-immune fraction, blood samples were obtained from a cohort of four patients with severe PAH. The detailed clinical, anthropometric, and hemodynamic characteristics of this study cohort are summarized in Table S1. Using a targeted density-gradient approach [17], we successfully isolated adherent cells from the wedged pulmonary arterial blood. On the basis of the transcriptional features described in Section 2.2, these are referred to as PAH MAC-like adherent cultures, abbreviated PAH MACs for readability throughout the remainder of the manuscript. The isolated cells exhibited an endothelial-like cobblestone morphology upon reaching confluence (days 16–20), structurally resembling reference human umbilical vein endothelial cells (HUVECs) (Figure 1a,b).

The lineage features of the isolated cells were verified prior to RNA sequencing using confocal immunocytochemistry. The cells stained positive for the intracellular endothelial marker von Willebrand factor (vWF), which localized distinctly to Weibel-Palade bodies. The cultures were negative for alpha-smooth muscle actin (ACTA2) (Figure 1c,d). ACTA2 negativity excludes contamination by smooth muscle cells and myofibroblasts, but it does not exclude cells of monocyte or macrophage origin; the lineage composition of the cultures was therefore examined transcriptomically, as described below.

### 2.2. Lineage Composition of the Isolated Adherent Cultures

To define the lineage composition of the isolated adherent cells, we examined canonical lineage markers in the RNA-seq data before global differential expression analysis. Endothelial transcripts were expressed at levels comparable to those of both endothelial reference populations and were not identified as differentially expressed in any pairwise comparison at the thresholds used (|log2FC| > 1, adjusted *p* < 0.05); these included *CDH5* (VE-cadherin), *PECAM1*, *VWF*, *FLT1* (VEGFR-1), *KDR*, *TEK* and *CLDN5*, as well as the endothelial-specifying transcription factors *ERG*, *FLI1* and *SOX17* (Table S4, Figure S5). In parallel, the same cultures expressed high levels of transcripts that were essentially absent from both endothelial references, including the pan-leukocyte marker *PTPRC* (CD45); the monocyte and macrophage markers *CD14*, *CD68*, *CD163*, *CSF1R*, *MSR1* and *LYZ*; and the myeloid-specifying transcription factors *SPI1* (PU.1), *IRF8* and *MAFB* (log2FC between 2.4 and 16.4 relative to HPAEC and HUVEC, adjusted *p* < 0.05).

This combination is consistent with the transcriptional characteristics described for myeloid angiogenic cells, that is, blood-derived mononuclear cells that acquire endothelial-like features. Accordingly, we refer to these cells as PAH MAC-like adherent cultures, abbreviated PAH MACs for readability. This terminology reflects the fact that the MAC identity was inferred from lineage-associated transcriptional features in bulk RNA sequencing rather than confirmed at the single-cell level; the abbreviation is used for convenience and does not imply cell-level confirmation of MAC identity. Two qualifications should be stated explicitly. First, the myeloid component of these cultures is not restricted to individual surface markers but includes mononuclear phagocyte transcription factors and mature macrophage-associated genes, indicating that the dominant transcriptional identity of the cultures is that of a mononuclear phagocyte rather than that of quiescent endothelium. Second, bulk RNA sequencing measures the average transcriptome of the culture and cannot establish whether endothelial and myeloid transcripts are co-expressed within individual cells or derive from two coexisting populations. We note that the endothelial program showed no reduction relative to the endothelial reference cultures, which would be expected if a large fraction of the culture were of non-endothelial origin, but this argument is indirect and a mixed composition cannot be formally excluded without cell-level analysis (see Limitations).

### 2.3. Transcriptomic Comparison of PAH MACs with Endothelial Reference Cultures

**Figure 2:**
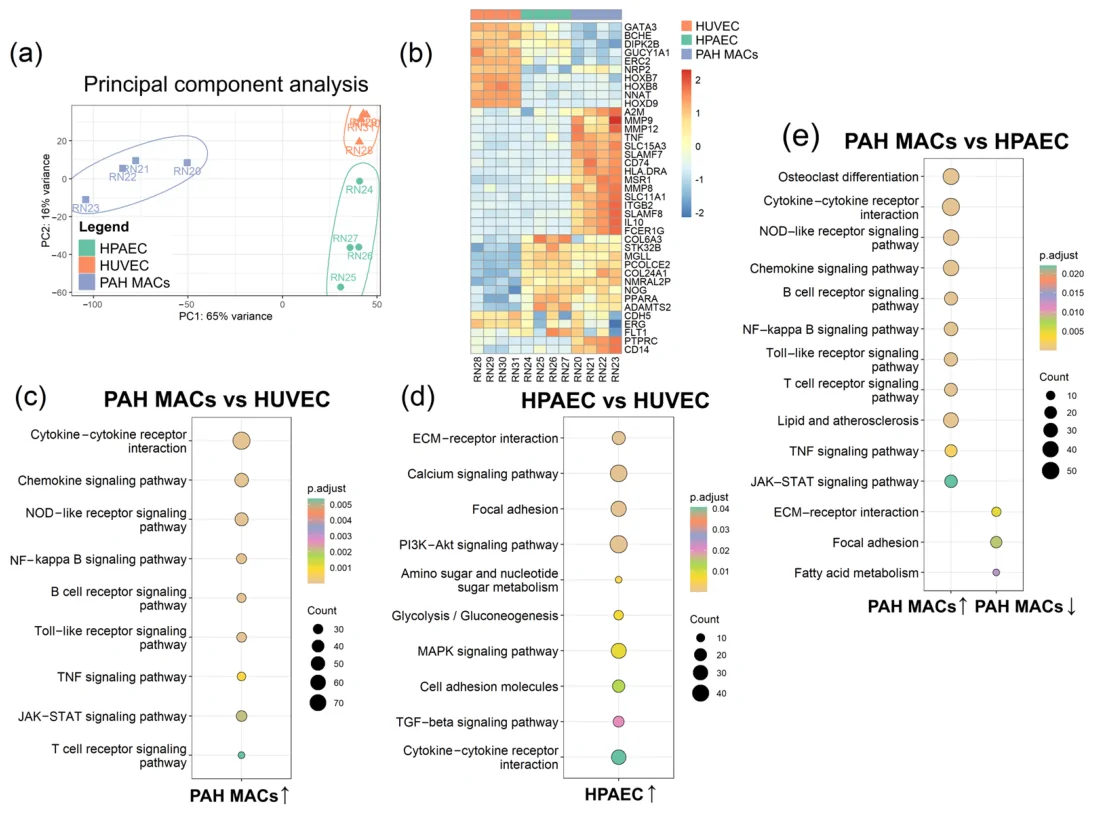
Differences in the transcriptomic profiles of the three cell populations studied. PAH MACs (purple)—adherent cultures isolated from wedged pulmonary arterial blood of patients with pulmonary arterial hypertension; HPAEC (green)—human pulmonary artery endothelial cells; HUVEC (orange)—reference endothelial cells. (**a**) Principal Component Analysis (PCA) plot. (**b**) Heatmap showing hierarchical clustering of selected differentially expressed genes (|log2FC| > 1, adjusted *p* < 0.05) across the samples. The color scale represents row Z-scores of normalized counts, indicating relative gene expression levels. (**c**) KEGG pathway enrichment for genes upregulated in PAH MACs relative to HUVEC. (**d**) KEGG pathway enrichment for genes upregulated in HPAEC relative to HUVEC. (**e**) KEGG pathway enrichment for genes upregulated in PAH MACs relative to HPAEC. Sample information is provided in Tables S1 and S2.

To characterize these cells at the transcriptome level, we performed bulk RNA sequencing on 12 independent samples: MACs from four PAH patients (*n* = 4 independent donors), human pulmonary artery endothelial cells (HPAEC) as a tissue-matched endothelial reference (*n* = 4 independent donors), and HUVEC as an additional endothelial reference (*n* = 4 independent donors). Principal Component Analysis demonstrated a clear segregation of the three cell types, with PAH MACs forming a distinct and separate cluster from both HPAEC and HUVEC, indicating a significant transcriptomic shift (Figure 2a).

Differential expression analysis (|log2FC| > 1, adjusted *p* < 0.05) identified extensive transcriptional differences between PAH MACs and both endothelial reference populations (Tables S2 and S3; Figures S1–S3). Because HPAEC is the tissue-matched reference, values for the PAH MACs versus HPAEC comparison are given first, with the HUVEC comparison provided in parentheses. Relative to HPAEC, PAH MACs showed higher transcript levels of classical pro-inflammatory cytokines and chemokines, including *IL1A* (log2FC = 3.74; vs. HUVEC 6.09), *TNF* (log2FC = 5.34; vs. HUVEC 8.01), *IL15* (log2FC = 1.25; vs. HUVEC 2.24) and *CCL2* (log2FC = 2.09; vs. HUVEC 2.56) (Figure 2b). The immunoregulatory cytokine *IL10* was also markedly higher (log2FC = 9.40; vs. HUVEC 11.91). Fold changes in this magnitude (*IL10*, *MMP12*) arise because the corresponding transcripts are close to the detection limit in the endothelial reference cultures, and they reflect the difference in lineage between the compared populations rather than graded activation of a common cell type. The corresponding absolute expression levels are moderate and are provided in Table S2. The expression of vascular cell adhesion molecule 1 (*VCAM1*) was also increased (log2FC = 1.83; vs. HUVEC 2.64). PAH MACs also expressed classical myeloid and phagocytic markers, including *CD33* and components of the complement system (*C1QA, C1QB*), which were essentially absent from both endothelial references. In addition, PAH MACs showed marked induction of matrix metalloproteinases *MMP8* (log2FC = 8.63, adjusted *p* = 2.04 × 10^−9^; vs. HUVEC 7.06, adjusted *p* = 3.15 × 10^−9^), *MMP9* (log2FC = 10.03, adjusted *p* = 1.33 × 10^−14^; vs. HUVEC 10.80, adjusted *p* = 9.16 × 10^−17^) and *MMP12* (log2FC = 8.58, adjusted *p* = 5.24 × 10^−9^; vs. HUVEC 11.94, adjusted *p* = 2.69 × 10^−12^), suggesting enhanced extracellular matrix turnover and tissue remodeling. *NOG* was elevated to a comparable extent in PAH MACs and in HPAEC relative to HUVEC (log2FC = 3.29 and 3.39, respectively) and did not differ between PAH MACs and HPAEC, indicating a feature shared with pulmonary endothelial cells rather than a PAH MAC-specific change. Overall, the gene-level differences indicate that PAH MACs display an inflammatory transcriptional profile associated with immune-cell recruitment and extracellular matrix remodeling.

Consistent with these gene-level changes, enrichment analysis revealed activation of immune and inflammatory pathways in PAH MACs compared with HUVEC and HPAEC. These included chemokine signaling, cytokine–cytokine receptor interaction, TNF, NOD-like receptor, NF-κB, Toll-like receptor, B-cell receptor, T-cell receptor, and JAK-STAT signaling pathways, indicating coordinated activation of both innate and adaptive immune-associated programs (Figure 2c,e). Together, these findings indicate that PAH MACs differ substantially from conventional HUVEC/HPAEC, exhibiting a transcriptional state characterized by inflammatory activation, enhanced matrix remodeling, and disruption of pathways supporting stable endothelial organization.

Comparison of HPAEC with HUVEC revealed differences consistent with the different tissue origins of the two endothelial references. At the level of individual genes, HPAEC showed increased expression of *VEGFA* (log2FC = 1.52, adjusted *p* = 0.02), *NOG* (log2FC = 3.39, adjusted *p* = 6.53 × 10^−7^), and *PPARA* (log2FC = 1.32, adjusted *p* = 6.81 × 10^−5^) (Figure 2b). Unlike PAH MACs, HPAEC did not show pronounced expression of inflammatory cytokines or immune-associated genes. In particular, *VEGFA* is consistent with active angiogenic signaling, while *PPARA* may contribute to lipid metabolic regulation and suppression of inflammatory transcriptional responses. The elevated expression of *NOG* may additionally reflect regulation of vascular growth and BMP signaling.

Pathway enrichment further supported the distinct characteristics of HPAEC. Compared with HUVEC, HPAEC were enriched for ECM-receptor interaction, focal adhesion, calcium signaling, PI3K-Akt, MAPK, TGF-β signaling, and glycolysis/gluconeogenesis (Figure 2d). These pathways relate to cell–matrix interactions, barrier organization, metabolic activity and angiogenic signaling. We note that the HPAEC cultures also expressed higher levels of mesenchymal transcripts than HUVECs, including *COL1A1*, *PDGFRB*, *ACTA2* and *SMTN*, consistent with the partial mesenchymal drift commonly observed in primary endothelial cultures. The enrichment of matrix- and adhesion-related pathways in this comparison should be interpreted with this in mind. Thus, while HPAECs retain stable structural, metabolic, and vascular signaling programs, PAH MACs exhibit a contrasting inflammatory and remodeling-associated state. When compared directly with HPAEC, PAH MACs showed lower expression of *HEYL* (log2FC = −6.16, adjusted *p* = 2.73 × 10^−5^) and *DLL4* (log2FC = −2.56, adjusted *p* = 1.87 × 10^−10^). These values are consistent with reduced endothelial Notch-associated transcriptional activity in PAH MACs relative to tissue-derived pulmonary endothelium. *HEYL* was also close to the detection limit in HUVEC, so its high level appears to be a property of the HPAEC cultures rather than a program lost by PAH MACs, and the functional significance of these differences for endothelial-to-smooth muscle interactions was not tested in this study. Taken together, PAH MACs differed from both endothelial reference populations far more than the two endothelial references differed from each other, and these differences were dominated by immune, chemokine and matrix-remodeling programs, in line with their myeloid transcriptional identity.

### 2.4. Protein–Protein Interaction Analysis Identifies Chemokine and Complement-Associated Networks in PAH MACs

**Figure 3:**
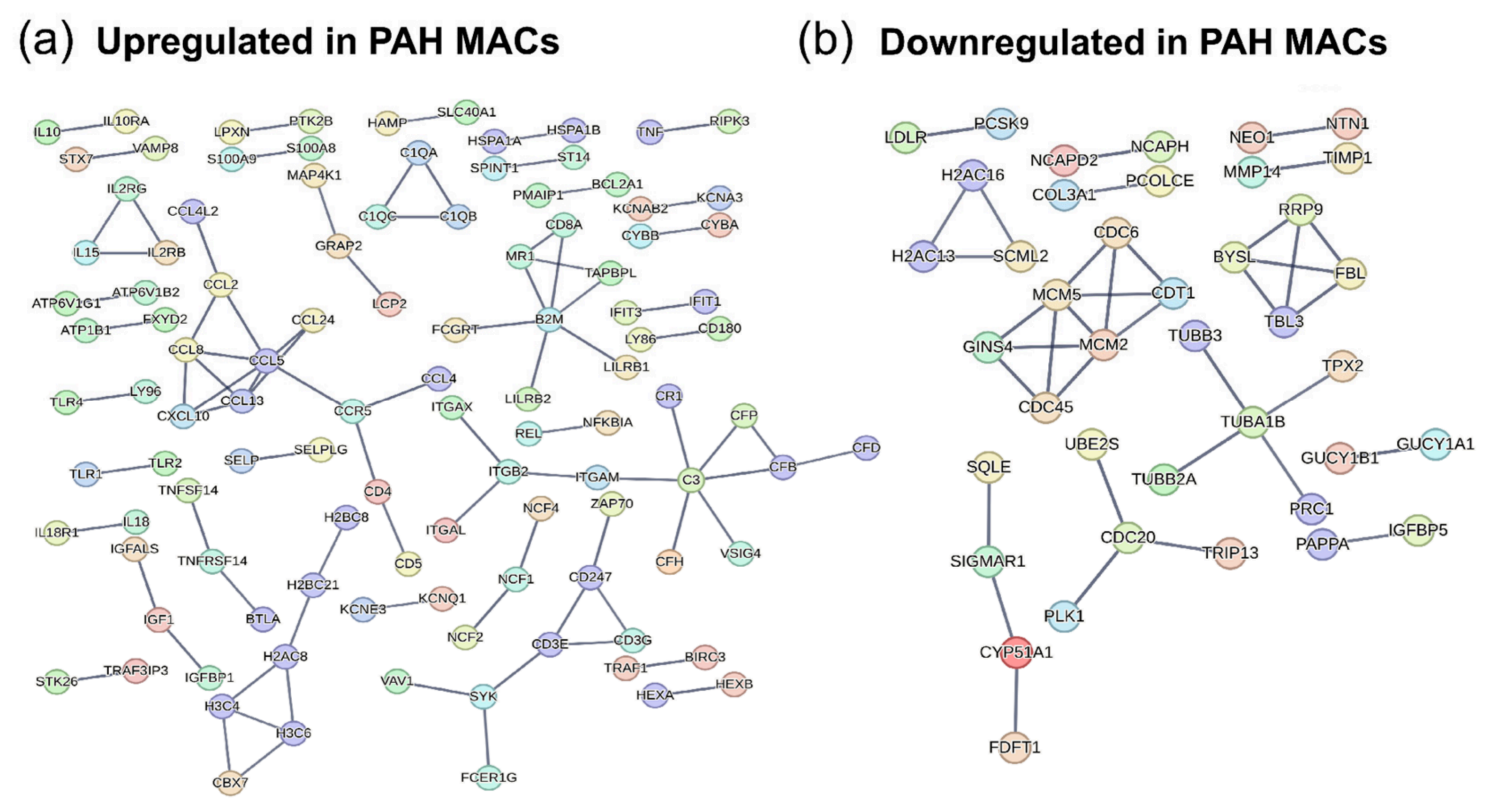
Protein–protein interaction network of proteins encoded by differentially expressed genes (DEGs) up- or downregulated in adherent cells isolated from wedged pulmonary arterial blood (PAH MACs) in comparison with pulmonary artery endothelial cells (HPAEC). (**a**) Network of proteins encoded by upregulated DEGs. (**b**) Network of proteins encoded by downregulated DEGs. Nodes represent individual proteins, and the connecting edges indicate experimentally validated functional interactions. Disconnected nodes are hidden. Minimum required interaction score = 0.900.

To further characterize the molecular alterations observed in PAH MACs relative to HPAEC, we performed protein–protein interaction network analysis using the STRING database. Separate networks were generated for proteins encoded by upregulated and downregulated differentially expressed genes (|log2FC| > 1 and adjusted *p* < 0.05) in PAH MACs relative to HPAEC. To focus on experimentally supported interactions, only experimental evidence was considered, with the highest confidence interaction score (≥0.900). Disconnected nodes were excluded from visualization. Under these criteria, the upregulated proteins formed several interconnected functional modules, whereas the downregulated proteins were predominantly organized into a distinct cell cycle-associated network (Figure 3).

**Figure 4:**
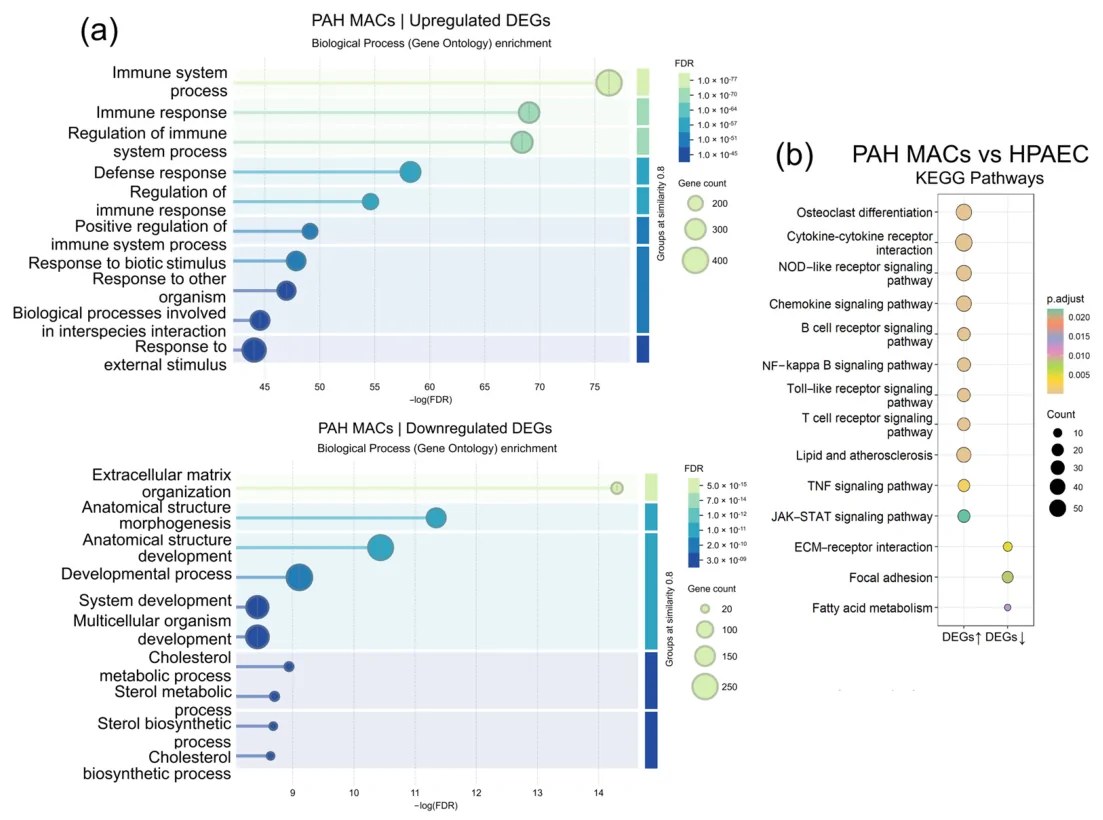
Functional enrichment analysis of differentially expressed genes (DEGs) up- or downregulated in adherent cells isolated from wedged pulmonary arterial blood (PAH MACs) in comparison with pulmonary artery endothelial cells (HPAEC). (**a**) Biological Processes Gene Ontology analysis. (**b**) KEGG pathway analysis.

The upregulated network was characterized by a prominent chemokine-centered interaction module, including CCL2, CCL4, CCL5, CCL8, CCL13, CCL24, and CXCL10 (Figure 3a). Among these, CCL5, CCL8, and CCL13 occupied central positions within the inflammatory interaction network. This coordinated enrichment of chemokine-associated proteins suggests that PAH MACs acquire an enhanced capacity for inflammatory signaling and leukocyte recruitment rather than exhibiting isolated changes in individual inflammatory genes. The network also contained a distinct complement-associated group involving C3, CFB, CFH, CFD, and CFP, with C3 representing a central connecting node. Together, these findings support activation of both chemokine-mediated inflammatory signaling and complement-associated immune processes in PAH MACs. Functional enrichment analysis of up- and downregulated DEGs independently confirmed the robust activation of these immune-related pathways (Figure 4a,b).

In parallel, the network of upregulated proteins contained a group of proteins characteristic of myeloid immune signaling, including FCER1G, SYK, LILRB1, LILRB2, ITGAX, LY86, CD180, and VSIG4. Notably, FCER1G and SYK were integrated into a broader signaling network involving integrin-associated proteins, while LILRB1/LILRB2 further supported an immunoregulatory myeloid phenotype. Thus, the network architecture extends beyond a generalized inflammatory response and points toward coordinated acquisition of myeloid-like signaling features by PAH MACs. In contrast, the downregulated network was dominated by proteins involved in DNA replication and cell-cycle progression, including MCM2, MCM5, CDC6, CDC45, CDC20, PLK1, and TPX2, together with tubulin-associated proteins (Figure 3b). This pattern indicates attenuation of proliferative and cell-cycle-associated molecular programs in PAH MACs compared with HPAEC.

Overall, the analysis revealed a coordinated molecular shift in PAH MACs characterized by enhanced chemokine and complement-associated immune signaling together with the acquisition of myeloid-like signaling features and reduced cell-cycle activity. The convergence of the CCL/CXCL chemokine network, complement components, and myeloid immunoreceptor signaling provides molecular support for the proposed immunomodulatory phenotype of PAH MACs. These transcriptional features are compatible with a capacity for leukocyte recruitment and inflammatory signaling within the pulmonary vascular microenvironment. This capacity was predicted from transcript abundance and network topology and was not tested functionally in the present study.

## 3. Discussion

The present study provides a novel transcriptomic characterization of myeloid angiogenic cells (MACs) isolated directly from the pulmonary arterial circulation of patients with PAH. Historically referred to as “early endothelial progenitor cells”, these mononuclear-derived adherent cells acquire endothelial-like morphology and express classical endothelial markers (such as vWF, VE-cadherin, and ERG) while retaining hematopoietic markers like CD45 and CD14. In a healthy physiological state, these cells are recruited to sites of vascular denudation to promote endothelial regeneration and angiogenesis. In the present study, cultures obtained from the pulmonary arterial blood of patients with PAH showed a transcriptional profile dominated by inflammatory and matrix-remodeling programs rather than by the programs of quiescent endothelium.

PAH MACs sampled from the wedged pulmonary artery carried a transcriptional signature composed of inflammatory mediators (*IL1A, TNF*), matrix metalloproteinases (*MMP8*, *MMP9*, *MMP12*) and myeloid signaling molecules (*TREM2*, *SYK*, *C1QA* and *C1QB*). Because MACs from non-PAH subjects were not available, we cannot determine which part of this signature is attributable to the PAH microenvironment and which reflects the intrinsic biology of monocyte-derived cells maintained under these culture conditions. Both contributions are plausible and are not separated by the present design. *IL10* transcript was also detected and was essentially absent from both endothelial references; *IL10* expression alone is not sufficient to assign a macrophage polarization state, particularly given the recognized limitations of the M1 and M2 framework, and we therefore do not interpret it as evidence of alternative activation. Similarly, whether these cells contribute to matrix turnover or to leukocyte recruitment in vivo was not addressed here, and the CCL5, CCL8 and CCL13 modules identified in the network analysis are transcriptional predictions that require functional testing.

While recent studies have described disease-associated defects in circulating endothelial cells isolated from peripheral venous blood [14], our approach captures adherent cells directly from the wedged pulmonary artery, situating the cellular phenotype closer to the primary site of pathology. Our transcriptomic lineage analysis extends this work by showing that such cultures are not purely endothelial and are dominated by cells with a mononuclear phagocyte transcriptional program. This transcriptomic shift provides a crucial mechanistic explanation for previously reported functional deficits. While earlier studies, historically classifying this population as early endothelial progenitor cells, demonstrated that circulating angiogenic cells in PAH exhibit impaired migratory, adhesive, and reparative capacities [15,16], our data suggest that the cultures used in these earlier studies are likely to have carried a substantial myeloid inflammatory component, which should be taken into account when functional deficits are attributed to endothelial progenitors.

Methodologically, this study reinforces a critical limitation in current pulmonary vascular sampling techniques. As we previously reported, attempts to isolate endothelial cells directly from the balloon surface of Swan-Ganz catheters were entirely unsuccessful across 19 independent procedures [17]. While balloon-harvesting has been proposed by others as a source of pure in situ PAECs [5,12], our consistent failure to obtain cells using this approach highlights its low reproducibility in our hands. In contrast, isolating adherent cells from the blood drawn directly from the wedged pulmonary artery proved to be a robust alternative [17]. Importantly, our current transcriptomic data now reveal a crucial caveat to this alternative: rather than yielding quiescent endothelium, this method selects for highly active myeloid angiogenic cells. Recognizing this myeloid inflammatory signature is essential for any future study that uses patient-derived circulating cell models. We also note that these cells were obtained from blood aspirated during balloon wedging and not from the pulmonary artery wall. We therefore describe them as MACs isolated from wedged pulmonary arterial blood and make no claim regarding a pulmonary arterial tissue origin; they may equally represent circulating or recruited cells sampled from the pulmonary vascular compartment.

Our PPI network analysis revealed the emergence of specific macrophage-like signaling hubs. Specifically, we identified a chemokine module centered on CCL5, CCL8 and CCL13. Chemokines of this family have been reported to mediate perivascular macrophage-smooth muscle cell interactions in PAH [8], although we did not test this in the present system. PAH MACs expressed components of the complement system (*C1QA*, *C1QB*) and classical phagocytic lineage markers (*CD33*, *SYK*), which is expected for cells of mononuclear phagocyte lineage and confirms the lineage assignment made in Section 2.2. SYK signaling regulates macrophage-like phenotypic switching and metabolic stress responses [4,8].

Genes associated with endothelial Notch signaling, *HEYL* and *DLL4*, were expressed at lower levels in PAH MACs than in HPAEC. The Notch pathway is important for vascular stability [18,19], but transcript-level differences in these genes do not by themselves establish a failure of intercellular communication, particularly between populations of different lineage. Likewise, the elevated expression of *MMP8*, *MMP9* and *MMP12* indicates a capacity for matrix proteolysis and remodeling; it does not establish a profibrotic phenotype, which would require assessment of matrix synthesis and deposition. *NOG* was elevated in both PAH MACs and HPAEC relative to HUVEC and therefore does not distinguish PAH MACs from pulmonary endothelial cells. *PPARA*, a regulator of endothelial lipid homeostasis that can suppress inflammatory transcriptional responses, was expressed at higher levels in HPAEC than in HUVEC (log2FC = 1.32, adjusted *p* = 6.8 × 10^−5^).

Several limitations should be emphasized. First, and most importantly, no MAC population from healthy or non-PAH subjects was available for comparison. An equivalent control would require right heart catheterization with balloon wedging in individuals without a clinical indication for the procedure, which is not ethically justifiable, and MACs obtained from peripheral venous blood would originate from a different vascular compartment. The comparisons presented here are therefore between PAH-derived MACs and endothelial reference cells, that is, between populations of different lineage, and they do not establish that the inflammatory and matrix-remodeling profile is caused by PAH. Throughout the manuscript the findings are accordingly described as the transcriptomic phenotype of PAH-derived MACs relative to endothelial reference cells.

Second, bulk RNA sequencing reflects the average transcriptome of the culture. It cannot resolve heterogeneity within the population and cannot establish that endothelial and myeloid transcripts are co-expressed within the same cells. Dual or multiplex immunofluorescence, flow cytometry or single-cell RNA sequencing would be required for this. The patient-derived cultures were fully consumed during RNA isolation and equivalent material cannot be obtained retrospectively from the same procedures, so these analyses are left for future work.

Third, no orthogonal validation at the protein or functional level was performed. The conclusions are therefore restricted to transcript abundance, and wording describing cellular function has been avoided throughout.

Fourth, the cells were analyzed after 16 to 20 days of culture in endothelial growth medium. The contribution of the culture period itself to the inflammatory transcriptional state cannot be separated from that of the in vivo environment, and a short-term or uncultured comparator was not available.

Fifth, the cohort comprised four patients with different PAH etiologies (IPAH, SSc-PAH and CHD-PAH), a wide hemodynamic range and differing treatment status. With *n* = 4 the effects of etiology, disease severity, medication, age and sex cannot be separated from the reported transcriptomic phenotype, and the study is hypothesis-generating. Individual patient characteristics and the corresponding RNA-seq sample identifiers are given in Table S1, and the relationship between individual samples is shown by unsupervised clustering in Figure S4.

Sixth, detailed clinical data for the HPAEC tissue donors were not available to the investigators. In addition, the HPAEC cultures expressed higher levels of mesenchymal transcripts than HUVEC, including *COL1A1*, *PDGFRB*, *ACTA2* and *SMTN*, consistent with the partial mesenchymal drift commonly observed in primary endothelial cultures. Both points should be taken into account when the HPAEC comparison is interpreted, particularly for matrix- and adhesion-related pathways.

## 4. Materials and Methods

### 4.1. Study Population and Clinical Characteristics

The study cohort included four patients with severe PAH (three women and one man) admitted to the Almazov National Medical Research Centre. The cohort encompassed diverse clinical etiologies: idiopathic PAH (IPAH), systemic sclerosis-associated PAH (SSc-PAH), and congenital heart disease-associated PAH (CHD-PAH). All patients exhibited significant pulmonary vascular remodeling and severe hemodynamic compromise. Comprehensive clinical, anthropometric, and hemodynamic data were collected during routine diagnostic right heart catheterization (RHC). Individual baseline clinical, anthropometric and hemodynamic characteristics of each patient, together with the corresponding RNA-seq sample identifier, are given in Table S1.

### 4.2. Cell Isolation and Culture Protocols

Isolation of Adherent Cells from Wedged Pulmonary Arterial Blood (PAH MACs): The cells were isolated from blood drawn directly from the lumen of the pulmonary artery through the Swan-Ganz catheter during balloon wedging, utilizing a density-gradient protocol validated by our group [17]. Briefly, freshly collected pulmonary arterial blood was diluted 1:1 with phosphate-buffered saline (PBS) and layered over a Ficoll density gradient solution. Following centrifugation at 400× *g* for 40 min without a brake, the mononuclear cell fraction was harvested, washed, and centrifuged at 300× *g* for 10 min. The resulting cell pellet was resuspended in Endothelial Cell Growth Base Media (Cell Application Inc., San Diego, CA, USA) supplemented with 1% penicillin/streptomycin. Cells were seeded onto 6-well plates pre-coated with 0.2% gelatin and cultured at 37 °C in a humidified 5% CO_2_ atmosphere.

Control Cell Lines: Human pulmonary artery endothelial cells (HPAECs) were isolated from pulmonary artery tissue obtained from four independent donors during heart transplantation procedures. Detailed clinical data for these tissue donors were not available to the investigators, and the absence of donor clinical characterization is acknowledged as a limitation. The tissues were washed, incubated in Collagenase III solution for 30 min at 37 °C, mechanically dissociated, and cultured up to 70–80% confluence. Human umbilical vein endothelial cells (HUVECs) were isolated from four independent umbilical cords following the classic Jaffe protocol [20] and provided by the Almazov NMRC biobank (Almazov National Medical Research Centre, Saint Petersburg, Russia).

### 4.3. Immunocytochemistry

To confirm phenotypic features, cells were cultured on coverslips and fixed with 4% paraformaldehyde (PFA) for 30 min at room temperature. Following fixation, cells were permeabilized with 0.1% Triton X-100 for 10 min, washed with PBS, and blocked with 1% BSA for 1 h. The coverslips were incubated in a humidified chamber for 1 h at room temperature with primary antibodies: anti-vWF (1:250; rabbit polyclonal, cat. no. ab9378, Abcam, Cambridge, UK), and anti-α-SMA/ACTA2 (1:500; mouse monoclonal, clone 1A4, cat. no. A2547, Sigma-Aldrich, St. Louis, MO, USA). Cells were incubated with Alexa Fluor 555-conjugated anti-rabbit IgG (1:1000) and Alexa Fluor 488-conjugated anti-mouse IgG (1:1000) secondary antibodies (Invitrogen, Thermo Fisher Scientific, Waltham, MA, USA). Coverslips were mounted using a DAPI-containing mounting medium optimized for fluorescence microscopy. Imaging was performed using an Olympus FV3000 confocal laser scanning microscope (Olympus, Tokyo, Japan).

### 4.4. RNA Extraction, Sequencing, and Bioinformatics Pipeline

Total RNA was isolated from cultured cells using the ExtractRNA kit (Evrogen, Moscow, Russia) according to the manufacturer’s protocol. RNA concentration and purity were assessed on a NanoDrop 1000 spectrophotometer (Thermo Fisher Scientific, Waltham, MA, USA). Library preparation and sequencing were carried out by BMKGENE. Donor sex was additionally confirmed in silico from the expression of *XIST* and of the Y-chromosome genes *RPS4Y1*, *DDX3Y*, *KDM5D*, *UTY* and *EIF1AY*, which also verified that the four samples in each group originated from four different individuals.

RNA sequencing was performed by BMKGENE (https://www.bmkgene.com/, accessed on 19 August 2026) on the Illumina NovaSeq X platform (Illumina, San Diego, CA, USA) using a paired-end sequencing strategy, with a sequencing depth of approximately 40 million reads (150 bp) per sample. Bulk RNA sequencing was performed on 12 samples, comprising 4 PAH MAC, 4 HPAEC, and 4 HUVEC independent biological replicates, processed in a single batch. The quality of the raw sequencing data was assessed using FastQC [21] and MultiQC [22]. All 12 libraries passed the predefined sequencing quality-control criteria, and no samples were excluded from downstream analysis. Raw reads were subjected to adapter trimming, poly-A/G tail removal, and filtering of short reads (<30 bp) utilizing Cutadapt version 4.4 [23]. The cleaned reads were aligned to the indexed human reference genome (GRCh38, GENCODE) using STAR version 2.7.10b [24,25]. Read counting was performed with featureCounts version 2.0.8 [26].

Differential gene expression analysis was conducted in R version 4.5.2 (R Foundation for Statistical Computing, Vienna, Austria) using RStudio (Posit Software, PBC, Boston, MA, USA) [27] and the DESeq2 package, version 1.50.2 [28]. Log2 fold changes were shrunk using lfcShrink, and *p*-values were adjusted by the Benjamini–Hochberg procedure. Genes were considered differentially expressed based on thresholds of |log2FC| > 1 and an adjusted *p*-value < 0.05. Endothelial and myeloid lineage identity was verified in silico by evaluating the expression levels of canonical markers (*CDH5*, *ERG*, *FLT1*, *PTPRC*, *CD14*). Data visualization—including PCA plots and heatmaps—was generated using the ggplot2 [29] and pheatmap [30] libraries. Pathway enrichment analysis was performed using the KEGG database [31]. A Protein–Protein Interaction network was constructed using the STRING database [32].

## 5. Conclusions

Adherent cells cultured from blood aspirated at the wedged pulmonary artery of patients with severe PAH are dominated by a mononuclear phagocyte transcriptional program while retaining expression of canonical endothelial genes, and they carry a transcriptional signature of inflammatory mediators, CC chemokines, complement components and matrix metalloproteinases. Because MACs from non-PAH subjects were not available, these features describe the phenotype of PAH-derived MACs relative to endothelial reference cells and are not established as PAH-specific. The principal implication of this work is methodological: protocols that isolate adherent cells from pulmonary arterial blood should not be assumed to yield pulmonary artery endothelial cells, and the lineage composition of such cultures should be verified before they are used as patient-derived endothelial models. If confirmed in studies that include MACs from subjects without PAH, the chemokine and complement modules identified here would be plausible candidates for therapeutic strategies directed at the recruited myeloid compartment in PAH. Whether these programs are stable or reversible, and whether they can be modulated pharmacologically, are questions for studies that combine appropriate cellular controls, cell-level resolution and functional validation.

## Data Availability

The RNA-seq count matrix for all 12 samples and the complete results of the differential expression analysis are provided in Table S2. Raw sequencing data are not publicly available due to privacy restrictions and are available from the corresponding author upon reasonable request.

## Supplementary Materials

The following supporting information can be downloaded at: https://www.mdpi.com/article/10.3390/ijms27198799/s1.

## Abbreviations

The following abbreviations are used in this manuscript:

ACTA2	Alpha-smooth muscle actin
BMP	Bone morphogenetic protein
BSA	Bovine serum albumin
CECs	Circulating endothelial cells
CHD-PAH	Congenital heart disease-associated pulmonary arterial hypertension
DAPI	4′,6-diamidino-2-phenylindole
DEGs	Differentially expressed genes
EndoMT	Endothelial-to-mesenchymal transition
HPAECs	Human pulmonary artery endothelial cells
HUVECs	Human umbilical vein endothelial cells
IPAH	Idiopathic pulmonary arterial hypertension
KEGG	Kyoto Encyclopedia of Genes and Genomes
MACs	Myeloid angiogenic cells
PAECs	Pulmonary artery endothelial cells
PAH	Pulmonary arterial hypertension
PBS	Phosphate-buffered saline
PCA	Principal component analysis
PFA	Paraformaldehyde
PPI	Protein–protein interaction
RHC	Right heart catheterization
SSc-PAH	Systemic sclerosis-associated pulmonary arterial hypertension
TGF-β	Transforming growth factor beta
vWF	von Willebrand factor
