# Supplementary material for: Transcriptomic Profiling of Adherent Cells Isolated from Wedged Pulmonary Arterial Blood of Patients with Pulmonary Arterial Hypertension Reveals a Predominantly Myeloid Rather than Endothelial Phenotype
Source: Int J Mol Sci. 2026 Oct 1;27(19):8799. doi: 10.3390/ijms27198799 (PMC13665573; doi:10.3390/ijms27198799)
Supplement: Supplementary file 1 [file ijms-27-08799-s001.zip › Supplementary Materials.pdf]

Supplementary Materials

Table S3. Top-10 up- or downregulated DEGs

DEGs	PAH MACs vs HPAEC		DEGs	PAH MACs vs HUVEC		DEGs	HPAEC vs HUVEC	
	Log2FC	Adj. p		Log2FC	Adj. p		Log2FC	Adj. p
**CD74**	9.53	1.04e-52	**CD74**	9.37	3.04e-51	**TMEM132B**	7.47	4.79e-27
**HLA-DRA**	14.01	2.59e-46	**SLC15A3**	4.23	9.43e-47	**ADAMTS12**	7.25	3.26e-25
**ITGB2**	10.03	3.81e-41	**SLC11A1**	9.04	1.82e-46	**TGFBI**	8.51	9.25e-25
**SLC11A1**	8.11	9.48e-41	**FCER1G**	8.83	6.25e-44	**AFAP1L1**	2.17	2.79e-24
**FCER1G**	8.41	2.63e-40	**HLA.DRA**	14.75	2.84e-43	**ENSG00000301761**	8.08	1.17e-23
**SLC15A3**	3.78	1.54e-37	**SLAMF7**	11.44	2.96e-41	**THBS2**	8.94	4.49e-23
**SLAMF7**	15.63	5.17e-33	**ITGB2**	9.30	1.19e-36	**MGLL**	1.89	1.82e-22
**HLA-DMB**	9.16	9.45e-32	**SLAMF8**	8.70	8.32e-35	**PCOLCE2**	9.52	2.39e-22
**ENSG00000248993**	7.62	9.45e-32	**MSR1**	7.32	3.54e-34	**STK32B**	3.75	3.31e-22
**SIGLEC1**	11.37	1.70e-31	**NMRAL2P**	4.63	4.57e-34	**NMRAL2P**	3.74	6.05e-22
**ADAMTS12**	-7.49	2.55e-27	**NNAT**	-4.43	8.66e-56	**NNAT**	-4.29	5.72e-53
**GATA4**	-7.57	3.47e-26	**HOXB7**	-4.45	3.21e-28	**HOXB7**	-3.96	2.86e-22
**MAP4K4**	-1.45	5.27e-21	**ENSG00000237380**	-5.65	5.05e-25	**MVB12B**	-2.26	1.32e-20
**ENSG00000303417**	-2.01	3.25e-17	**DIPK2B**	-4.72	5.14e-21	**HOXD9**	-12.14	3.61e-20
**KRT19**	-3.83	6.91e-17	**GUCY1A1**	-7.47	2.32e-20	**ENSG00000225216**	-1.59	4.82e-19
**ALDOC**	-2.82	1.53e-15	**HOXB8**	-7.09	1.18e-18	**ENSG00000237380**	-8.99	5.29e-19
**CCDC8**	-7.50	1.54e-14	**ERC2**	-5.60	6.48e-17	**ANXA2R**	-2.88	1.61e-17
**ENSG00000267328**	-5.85	5.42e-14	**GATA3**	-4.39	8.10e-17	**LDLRAD4**	-4.46	3.92e-17
**SMTN**	-1.62	7.68e-14	**HOXD9**	-8.88	2.08e-16	**ENSG00000300615**	-3.34	1.04e-16

**COL1A1**	-9.10	2.24e-13	**BCHE**	-4.80	1.86e-15	**NRP2**	-1.64	1.25e-16

Table S4. Lineage-defining endothelial and myeloid markers

**Gene**	**PAH MACs vs HPAEC**		**PAH MACs vs HUVEC**		**HPAEC vs HUVEC**	
	Log2FC	Adj. p-value	Log2FC	Adj. p-value	Log2FC	Adj. p-value
**CDH5**	0.10	0.82	-0.26	0.52	-0.32	0.37
**ERG**	-0.02	0.96	-0.44	0.16	-0.37	0.23
**FLT1**	-0.30	0.42	0.002	0.998	0.27	0.47
**PTPRC**	**16.44**	**9.76e-12**	**9.38**	**4.04e-07**	-0.19	0.07
**CD14**	**7.22**	**7.91e-14**	**8.25**	**9.87e-18**	0.20	0.56
**SPI1**	**5.92**	**8.86e-26**	**6.03**	**8.94e-27**	0.04	0.95
**IRF8**	**10.97**	**2.21e-15**	**9.98**	**1.59e-14**	-0.10	0.73
**MAFB**	**4.47**	**7.68e-11**	**2.45**	**0.0004**	**-1.41**	**0.03**
**FLI1**	0.31	0.44	-0.18	0.72	-0.45	0.22
**SOX17**	-0.01	0.99	-0.14	0.82	-0.09	0.86
**PECAM1**	0.05	0.93	0.13	0.80	0.07	0.90
**VWF**	0.46	0.34	0.02	0.99	-0.35	0.42
**KDR**	0.32	0.45	0.42	0.37	0.04	0.95
**TEK**	0.38	0.30	-0.27	0.054	-0.62	0.07
**CLDN5**	-0.70	0.02	-0.40	0.26	0.27	0.43

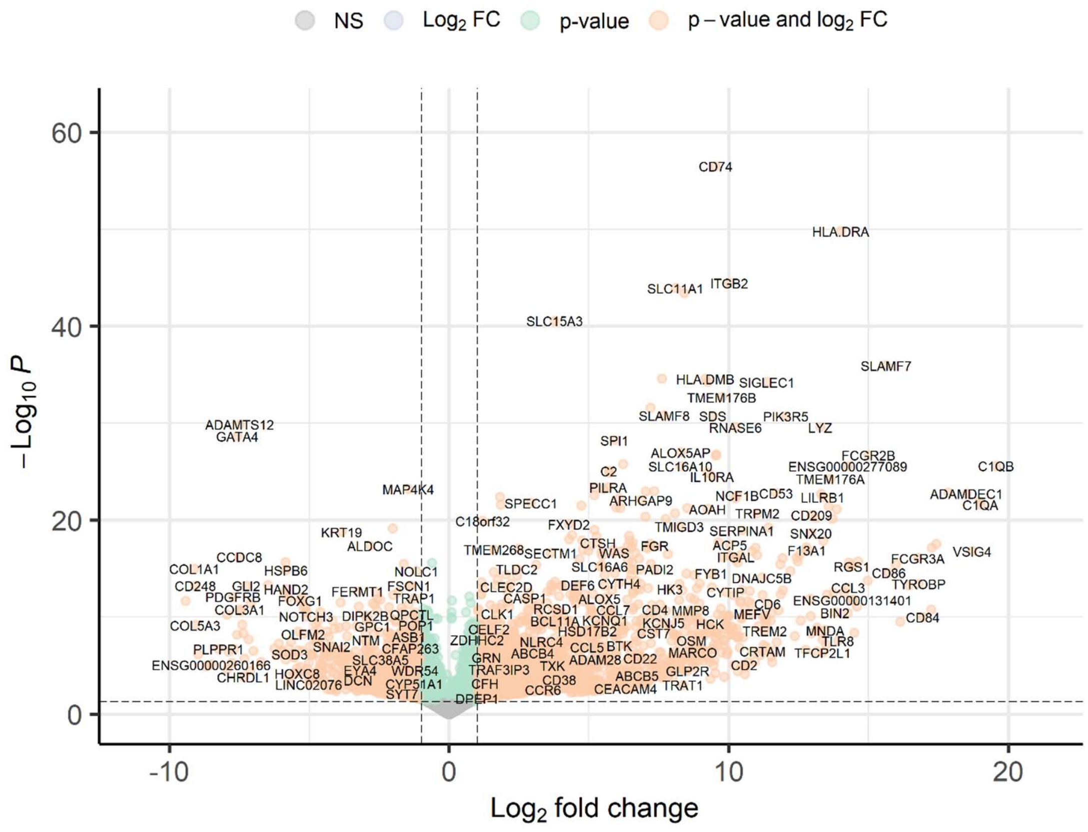

Figure S1. Volcano plot PAH MACs vs HPAEC.

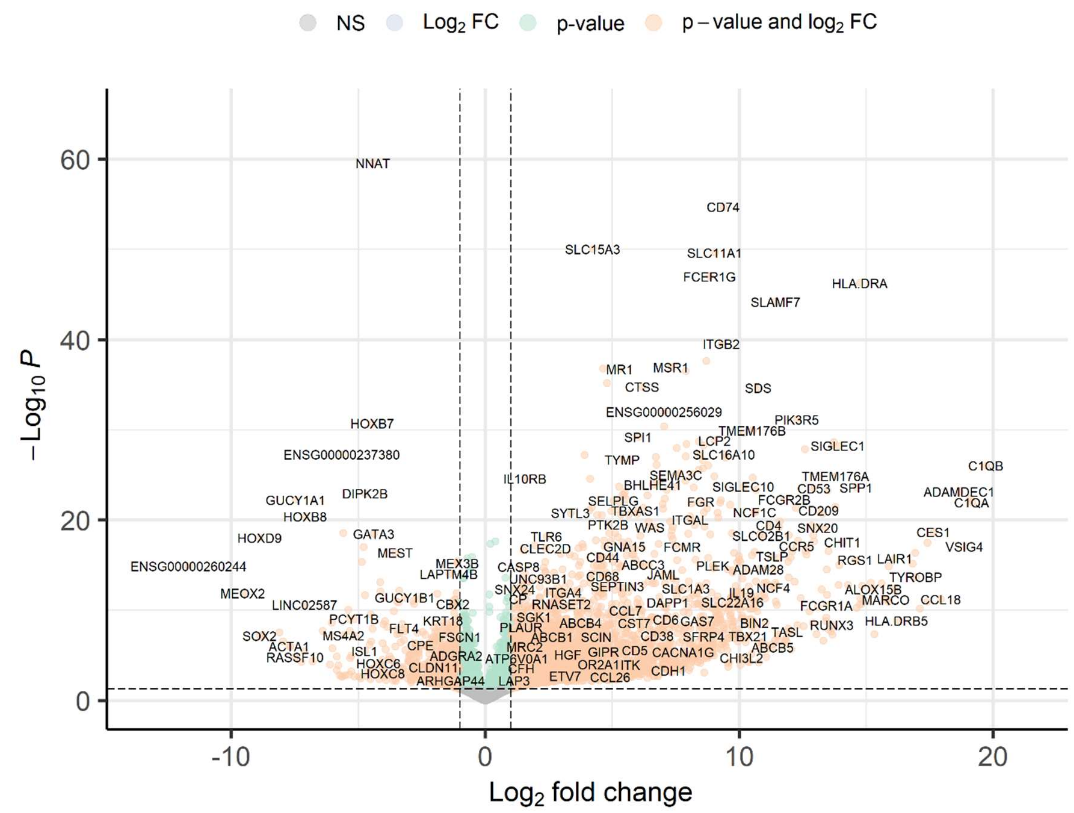

Figure S2. Volcano plot PAH MACs vs HUVEC.

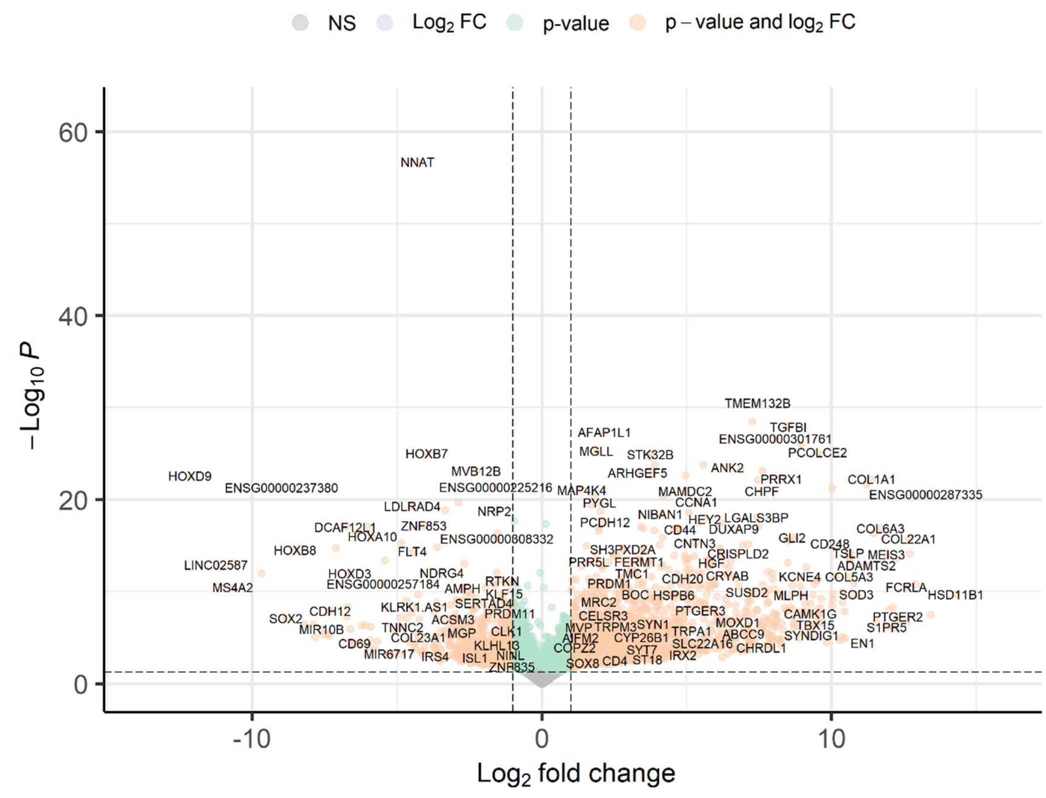

Figure S3. Volcano plot HPAEC vs HUVEC.

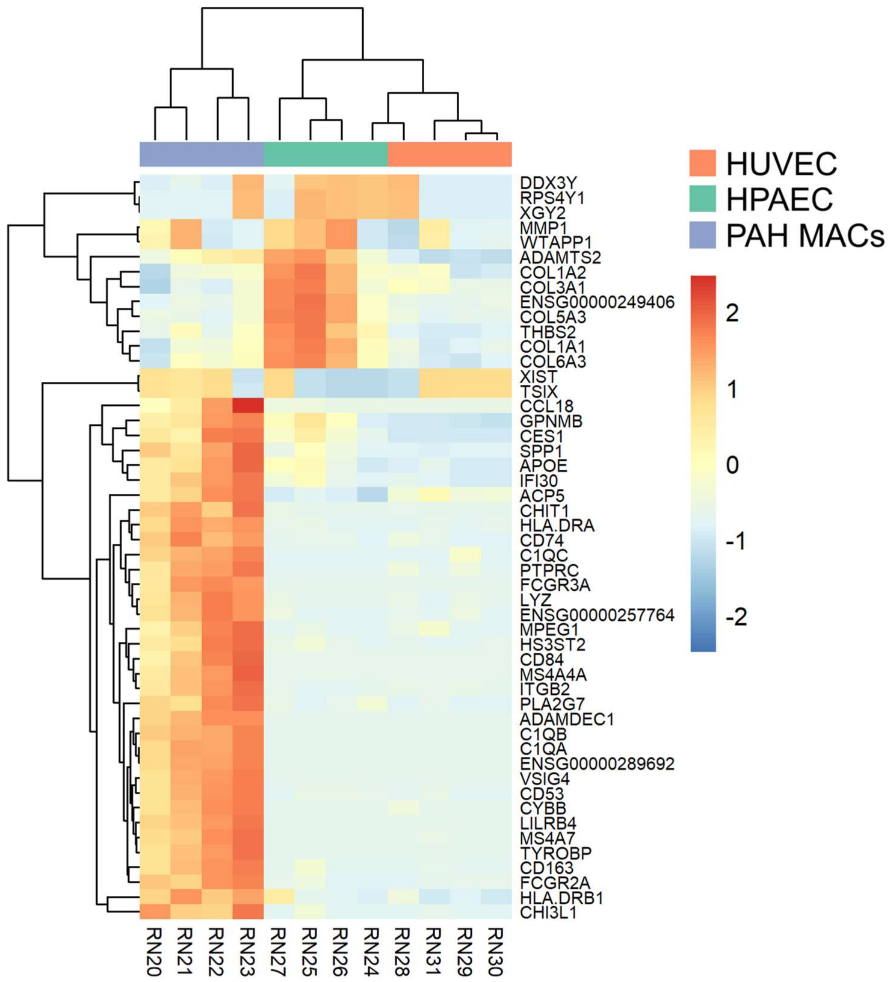

Figure S4. Unsupervised heatmap of the 50 most variable genes.

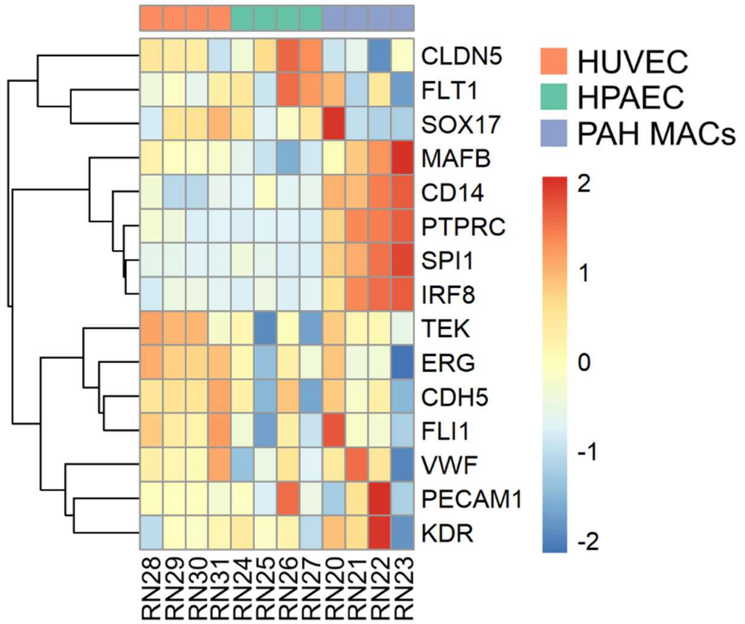

Figure S5. Heatmap of Lineage-defining endothelial and myeloid markers (Table S4).

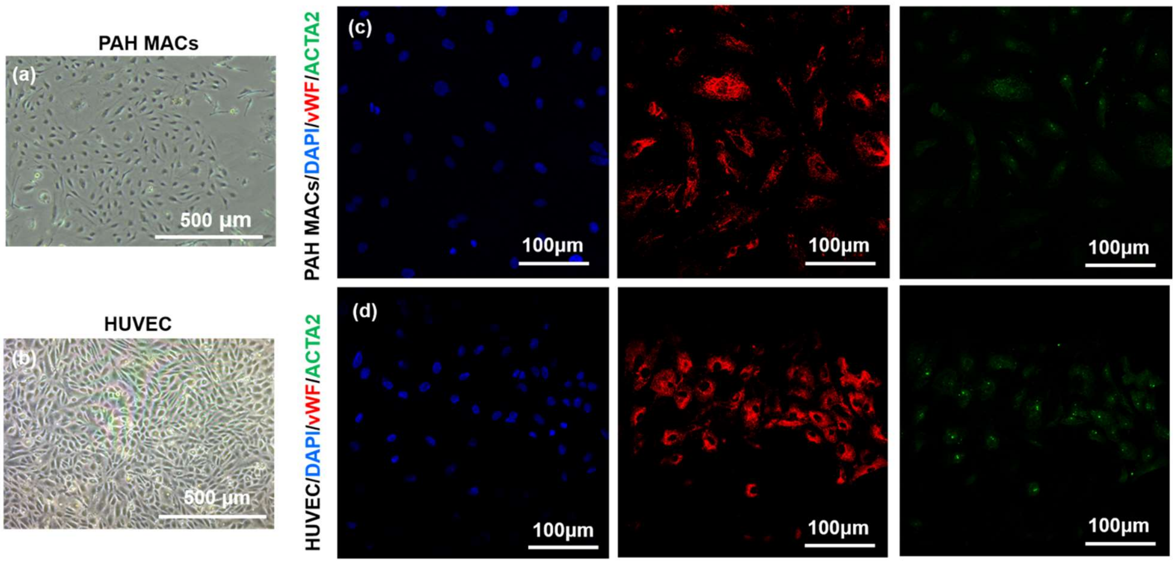

Figure S6. Single-channel immunofluorescence images of PAH MACs and HUVEC. DAPI (blue), vWF (red) and ACTA2 (green) channels are shown separately for the merged images presented in Figure 1c,d. Scale bars: 100 um.
