# Supplementary material for: Transcriptomic Profiling of Adherent Cells Isolated from Wedged Pulmonary Arterial Blood of Patients with Pulmonary Arterial Hypertension Reveals a Predominantly Myeloid Rather than Endothelial Phenotype
Source: Int J Mol Sci. 2026 Oct 1;27(19):8799. doi: 10.3390/ijms27198799 (PMC13665573; doi:10.3390/ijms27198799)
Supplement: Supplementary file 1 [file ijms-27-08799-s001.zip › Table S1 - Baseline clinical and hemodynamic characteristics.pdf]

Table S1. Baseline clinical and hemodynamic characteristics of the PAH study cohort.

Parameter	Patient 1	Patient 2	Patient 3	Patient 4
Sex	Female	Female	Female	Male
RNA-seq sample ID	U0001	U0002	U0003	U0004
**Demographics and Clinical Status**				
Age, years	40-50	60-70	30-40	30-40
Primary Etiology	IPAH (VR+)	SSc-PAH	IPAH	CHD-PAH (corrected PDA)
Comorbidities	None reported	CAD, HFpEF, HTN, Atherosclerosis, GERD	None reported	Chronic bronchitis, Obesity, Thrombocytopenia
WHO Functional Class	II	III	III	III
6MWD, m	418	373	410	300
Targeted PAH Therapy	Treatment-naïve	Treatment-naïve	Treatment-naïve	Riociguat, Macitentan
Diuretics	None	None	None	Torasemide, Spironolactone
BMI, kg/m²	31.6	20.6	27.7	31.2
BSA, m²	1.88	1.57	1.90	2.23
Survival Status*	Alive	Deceased	Alive	Alive
**Hemodynamics**				
SBP, mmHg	100	127	111	129
DBP, mmHg	55	73	67	97
mBP, mmHg	66	91	83	79
sPAP, mmHg	74	91	56	85
dPAP, mmHg	26	30	32	57
mPAP, mmHg	43	50	42	76
PAWP, mmHg	10	3	10	15
RAP/CVP, mmHg	18	1	4	11
HR, bpm	65	71	53	71

Parameter	Patient 1	Patient 2	Patient 3	Patient 4
CO, L/min	5.4	3.1	3.4	4.9
CI, L/min/m²	2.87	2.00	1.89	2.21
SV, mL	83.1	44.0	60.7	69.0
PVR, WU	5.4	15.0	9.4	12.4
PAC, mL/mmHg	1.73	0.72	2.53	2.46
SaO2, %	96.2	92.0	97.4	95.6
SvO2, %	69.8	57.0	56.3	70.4

Notes: *Survival status assessed as of August 11, 2026. Donor sex was confirmed *in silico* from XIST and Y-chromosome transcript expression in the RNA-seq data (Section 4.4). **Abbreviations: 6MWD**, 6-minute walk distance; **BMI**, body mass index; **BSA**, body surface area; **CAD**, coronary artery disease; **CHD-PAH**, congenital heart disease-associated pulmonary arterial hypertension; **CI**, cardiac index; **CO**, cardiac output; **CVP**, central venous pressure; **DBP**, diastolic blood pressure; **dPAP**, diastolic pulmonary arterial pressure; **GERD**, gastroesophageal reflux disease; **HFpEF**, heart failure with preserved ejection fraction; **HR**, heart rate; **HTN**, hypertension; **IPAH**, idiopathic pulmonary arterial hypertension; **mBP**, mean blood pressure; **mPAP**, mean pulmonary arterial pressure; **PAC**, pulmonary arterial compliance; **PAWP**, pulmonary arterial wedge pressure; **PDA**, patent ductus arteriosus; **PVR**, pulmonary vascular resistance; **RAP**, right atrial pressure; **SaO2**, arterial oxygen saturation; **SBP**, systolic blood pressure; **sPAP**, systolic pulmonary arterial pressure; **SSc-PAH**, systemic sclerosis-associated pulmonary arterial hypertension; **SV**, stroke volume; **SvO2**, mixed venous oxygen saturation; **VR+**, acute vasoreactivity test positive; **WHO**, World Health Organization; **WU**, Wood units.
